# Tau protein as a potential predictive marker in epithelial ovarian cancer patients treated with paclitaxel/platinum first-line chemotherapy

**DOI:** 10.1186/1756-9966-32-25

**Published:** 2013-04-30

**Authors:** Marta Smoter, Lubomir Bodnar, Bartlomiej Grala, Rafal Stec, Krystyna Zieniuk, Wojciech Kozlowski, Cezary Szczylik

**Affiliations:** 1 Oncology Department, Military Institute of Health Services in Warsaw, Szaserów Street 128, Warszawa, 04-141, Poland; 2 Pathology Department, Military Institute of Health Services in Warsaw, Szaserów Street 128, Warszawa, 04-141, Poland

**Keywords:** Tau protein, Ovarian cancer, Predictive factor, Prognostic factor, Chemotherapy

## Abstract

**Background:**

The aim of the study was to evaluate predictive and prognostic significance of microtubule-associated protein Tau in epithelial ovarian cancer (EOC) patients treated with paclitaxel and platinum-based chemotherapy.

**Methods:**

74 patients with EOC (stage I-IV) who underwent cytoreductive surgery followed by standard paclitaxel/platinum chemotherapy were included in the retrospective analysis. Their formalin-fixed, paraffin-embedded tissue specimens were immunohistochemically stained for Tau protein, using semi-quantitative DAKO test. Tau expression was acknowledged as negative (0 and 1+) or positive (2+ and 3+). The correlation between Tau expression, progression free survival (PFS) and overall survival (OS) was evaluated. Statistical analysis included Kaplan-Meyer estimator, long rank test, Mann Whitney test and Cox proportional hazards model.

**Results:**

25.7% (19/74) and 74.3% (55/74) of the patients were classified as Tau-negative and Tau-positive, respectively. Median PFS was 28.7 months for Tau-negative group and 15.9 months for Tau-positive group (p = 0.0355). In the univariate analysis 3-year OS in Tau-negative and Tau-positive groups was 80.2% and 52.4%, respectively (p = 0.0198). Low expression of protein Tau was associated with better OS, whereas an advanced stage at diagnosis, suboptimal surgery, serous histological type and resistance to first line chemotherapy were each correlated with worse OS (p <0,05). In multivariate analysis only resistance to first line chemotherapy remained significant (HR 22.59; 95% CI, 8.71-58.55; p <0.0001).

**Conclusions:**

Negative tau protein seems to be both good prognostic factor and a predictor of response to paclitaxel/platinum-based chemotherapy in EOC patients.

## Background

Ovarian cancer remains leading cause of death among patients with different gynecological neoplasms. Although majority of the patients respond to the primary treatment with debulking surgery followed by paclitaxel and platinum-based chemotherapy, many of them experience relapse of the disease within few years after first-line therapy.

Platinum compounds introduction to the ovarian cancer treatment was a corner stone in the therapy of this malignancy. Paclitaxel addition to platinum improves the results of chemotherapy [1,2]. Nevertheless about one quarter of the patients does not respond to the therapy and those who initially benefit from the treatment incline to experience disease recurrence.

There are no molecular agents known to predict the response to the chemotherapy in ovarian cancer as well as patients’ outcome. Revelation of such markers could result in a more effective patient selection to the certain regimens and development of tailored chemotherapy in ovarian cancer.

Recently, microtubule associated protein (MAP) Tau has been identified as a potential marker of response to paclitaxel in breast cancer. Tau protein (50–64 kD), a product of gene located in chromosome 17 (17q21) shows the ability of combining to beta-tubulin. It may bind to the exterior as well as to the interior microtubules surface, in the same binding site as paclitaxel, and consequently compete with this drug [3,4]. The loop of beta tubulin combined to Tau stabilizes microtubules in similar way as paclitaxel, but with a smaller affinity and greater reversibility [5]. Overexpression of Tau protein leads to increase of polymerization and at the same time reduces cells’ flexibility [6].

Six isoforms of Tau protein occur in nature and are divided into two groups, depending on the number of domains combined to tubulin. Tau-3L, Tau-3S and Tau-3 belong to group 3R and connects with tubulin by three domains, while Tau-4L, Tau-4S and Tau-4 (group 4R) uses four domains to bind to tubulin [7].

Tau protein activity and affinity to microtubules is regulated in phosphorylation processes by serine threonine kinases. Phosphorylation of certain places for example serine 262 or 396 is related to reduction of binding of Tau to microtubules [7]. At the same time, overphosphorylation of this protein leads to neurofibrillary degeneration and is suggested to have an important impact on pathogenesis of neurodegenerative diseases, which clinically demonstrate with the limitation of cognitive functions, including Alzheimer’s or Pick’s diseases [7].

Predictive or prognostic value of protein Tau in ovarian cancer has not been yet established. We aimed to determine the relevance of Tau expression in this malignancy. We have investigated retrospectively the correlation between immunohistochemical expression of protein Tau in the primary tumors and progression free survival (PFS) as well as overall survival (OS) in epithelial ovarian cancer patients treated with debulking surgery followed by standard paclitaxel/platinum chemotherapy.

## Materials and methods

### Patients

We included in our study consecutive patients treated in our site between March 2001 and December 2007, who fulfilled following inclusion criteria:

1) histologically confirmed epithelial ovarian cancer International Federation of Gynaecology and Obstetrics (FIGO) stage IC-IV,

2) history of debulking surgery followed by first-line chemotherapy regimen: paclitaxel (135 mg/m^2^) with cisplatin (75 mg/m^2^) or paclitaxel (175 mg/m^2^) with carboplatin (AUC6), administered every 3 weeks for 6 cycles,

3) accessibility of primary tumor specimens and full medical data.

Among 132 patients in our database, 74 were eligible. Remaining 58 patients were excluded from the analysis due to inaccessibility of primary tumour specimens (48), deficiency in clinical data (5) or diagnosis of concomitant malignancy (5). Table 1 summarizes clinical characteristics of the patients included in the analysis. Median age in the study group was 54 years (range 31–73). 79,7% of the patients was diagnosed at advanced FIGO stage (III-IV). Half of the patients had diagnosed serous type of ovarian cancer 64.9% of the group were sensitive to chemotherapy.

**Table 1:** Patient characteristics

**Median age, range (years)**	54 (31–73)
**Performance status (ECOG scale)**	
• 0	12.2% (9/74)
• 1	**81.1% (60/74)**
• 2	6.7% (5/74)
**Histologic cell type**	
• Serous	**50% (37/74)**
• Endometrioid	22.97% (17/74)
• Mucinous	6.76% (5/74)
• Clear cell	4.05% (3/74)
• Mixed	13.51% (10/74)
• Undifferentiated	1.35% (1/74)
• Others	1.35% (1/74)
**FIGO stage at diagnosis**	
▪ I	8.1% (6/74)
▪ II	12.2% (9/74)
▪ III	**58.1% (43/74)**
▪ IV	21.6% (16/74)
**Primary surgery**	
▪ Radical	16.2% (12/74)
▪ Optimal debulking	**48.6% (36/74)**
▪ Suboptimal debulking	35.1% (26/74)
**Grade (G)**	
▪ 1 and 2	41.9% (31/74)
▪ 3 and unknown	**58.1% (43/74)**
**Platinum sensitivity**	
Sensitive (>6 months)	**64.9% (48/74)**
Resistant (<6 months)	35.1% (26/74)

Local Research Ethics Committee approved the study on 19th of March 2008 (number 11/2008). Primary tumor specimens of the patients included in the analysis were immunohistochemically stained for tau protein. Patients’ data: response to first-line chemotherapy according to RECIST criteria, PFS, OS were obtained from medical records and retrospectively analyzed. Median observation period was 25 months (95% CI, 24–32).

### Immunochemistry

**Figure 1:**
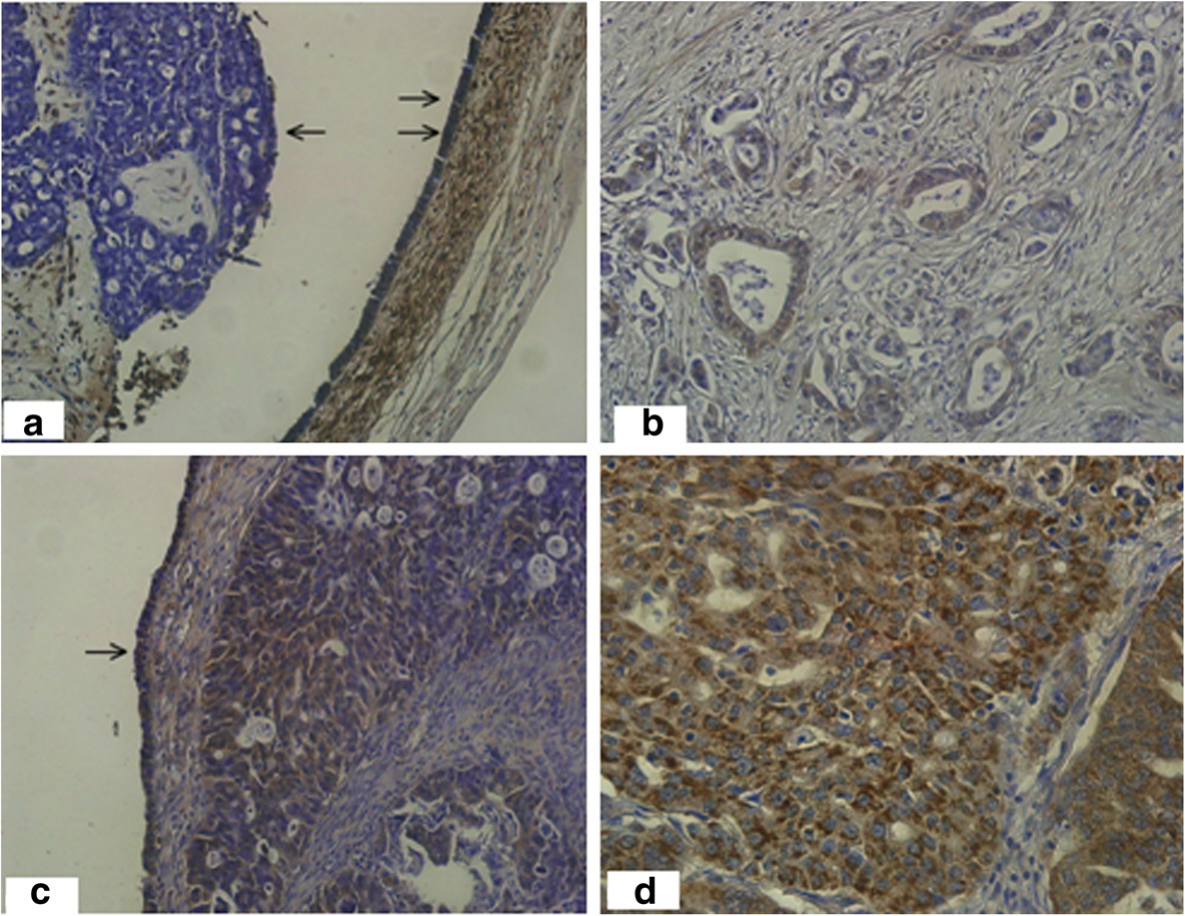
**Tau protein expression by IHC (a-d).** Tau 0 (**a**) - completely negative staining with anti-Tau antibody in tumor cells (left). Moderately intense staining of fallopian tube epithelium on the right (double arrows) serves as internal positive control (magnification 200×). Tau 1+ (**b**) Adenocarcinoma cells with weak focal expression of Tau protein (magnification 200×). Tau 2+ (**c**) Moderately intense staining of tumor cells similar to pattern of staining of superficial ovarian epithelium (arrow) (magnification 200×). Tau 3+ (**d**) Intense and diffuse staining as dark cytoplasmatic granules.

Material was obtained from primary tumors of 74 patients and immunohistochemically stained for Tau protein. In bilateral ovarian cancer cases (41/74), both tumors were stained. Formalin-fixed, paraffin-embedded 5-μm sections of ovarian cancer were incubated with anti-Tau polyclonal rabbit antibody that recognizes all isoforms of human Tau irrespectively of its phosphorylation status (1:100 dilution; code A 0024; DAKO Cytomation) for 30 minutes in room temperature. Anti-rabbit horseradish peroxidase-labeled secondary antibody was used to generate signal (code K 4002; DAKO Envision TM+ System). Normal ovarian epithelium derived from 51-year-old patient who had underwent surgery due to benign ovarian cyst was used as an external positive control. Omission of primary antibody served as a negative control. Specimens were assessed by means of light microscope with 20 × magnification lens. Tau staining of tumor cells was scored according to Rouzier et al. [4] with the authors’ modification as follows: IHC score 0 – no staining; 1+ − poor focal staining or very poor diffuse staining (less intense than normal ovarian epithelium); 2+ average diffuse staining (similar to normal ovarian epithelium) or strong staining (more intense than normal ovarian epithelium) in less than 25% cells; 3+ strong staining in 25% of tumors cells or more (Figure 1). Tau expression was acknowledged as negative (0 and 1+) or positive (2+ and 3+). This dichotomization of staining results was determined by using staining intensity of normal epithelial cells as a reference. In case of bilateral ovarian cancer the staining results from both ovaries were averaged. In case of averaged results, they were acknowledged as negative (0–1,5) and positive (2–3). Slides were scored without knowledge of the clinical outcome.

### Statistical analysis

Statistical analysis included descriptive statistics with determination of minimal and maximal values, means and medians, with 95% confidence interval (CI) for particular variables. The correlation between Tau expression and clinical parameters was assessed by X^2^ test. PFS was defined as the time from diagnosis until disease recurrence or death, while OS was the time from diagnosis until death or cut-off point which was 15 Dec 2009.

Analysis of PFS and OS was done by means of Kaplan-Meier method. Univariate analyses of variables influencing PFS or OS was performed by log-rank test, which identified preliminary list of significant factors. Multivariate analyses of PFS and OS were performed by Cox proportional-hazard regression using the forward stepwise method; all variables found to be significant in the univariate analysis were included in the multivariate analysis.

Statistical significance was defined as a probability level less than 0.05. Statistical calculation was performed using the STATISTICA for Windows Version 7.0 software.

## Results

### Tau expression in ovarian cancer

According to the best knowledge of the authors, in our study Tau expression was evaluated in ovarian cancer for the first time. Among 74 patients included in the analysis, 74.3% (n=55) were Tau-positive and 25.7% (n=19) were Tau-negative.

### Association between Tau expression and PFS

**Figure 2:**
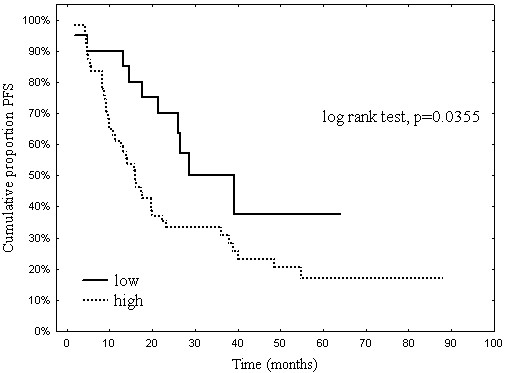
Progression free survival by tau expression.

Univariate analysis revealed following clinical parameters correlated with PFS: FIGO stage at diagnosis (p=0.004), ovarian cancer type (serous vs. others; p=0.0202), residual tumor size after debulking surgery (p=0.005) and tau expression level (p=0.0355). Age, performance status and tumor grade were not correlated with PFS. The results are presented in Table 2 and Figure 2.

**Table 2:** Univariate analysis of PFS ( log-rank test)

**Clinical parameter**	**n (% )**	**Median (months)**	**P value**
Age			0.3447
○ < 65	60 (81.1%)	17.4	
○ > 65	14 (18.9%)	20.0	
FIGO stage at diagnosis			
○ Early (I,II)	15 (20.3%)	76.3%†	0.0040*
○ Advanced (III,IV)	59 (79.7%)	33.3%†	
Histopathologic cell type			
○ serous	37 (50%)	16.8	0.0202*
○ others	37 (50%)	31.5	
Residual tumor size			0.0005*
○ <1 cm	48 (64.9%)	28.3	
○ > 1 cm	26 (35.1%)	8.9	
Performance status (ECOG)			0.1388
○ 0-1	69 (93.2%)	20.0	
○ 2	5 (6.7%)	17.4	
Tumor grade			0.4788
○ G1,G2	31 (41.9%)	26.7	
○ G3, unknown	43 (58.1%)	16.6	
Tau expression			0.0355*
○ negative	19 (25.6%)	28.7	
○ positive	55 (74.3%)	15.9	

†− if median was not achieved, the results were described as a percentage of patients with 2 years PFS *- statistical significance.

Multivariate analysis showed that among factors correlated with PFS in univariate analysis, only residual tumor size occurred as independently associated with PFS (p=0.0002, HR – 2.84) (Table 3).

**Table 3:** Multivariate analysis of PFS association with clinical parameters

**Clinical parameter**	**Multivariate analysis**	
	**HR (95% CI)**	**P value**
Tau expression		
onegative	NS	NS
opositive		
FIGO stage at diagnosis		
oEarly (I,II)	NS	NS
oAdvanced (III,IV)		
Histopathologic cell type		
oserous	NS	NS
oothers		
Residual tumor size		
o<1 cm	**2.84; (1.64-4.92)**	**0.0002**
o> 1 cm		

Abbreviations: HR- hazard ratio, NS – not significant.

### Association between Tau expression and OS

**Figure 3:**
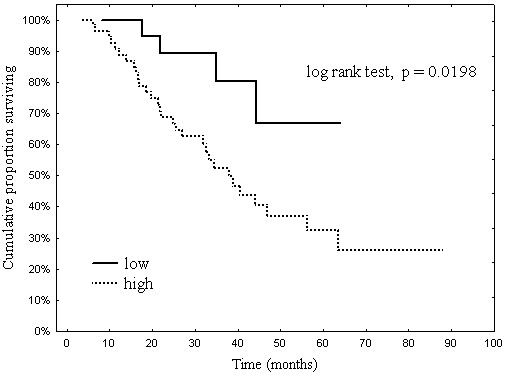
Overall survival by tau expression.

Clinical parameters correlated with OS were identified in univariate analysis and presented in Table 4. Statistical significance was achieved in following factors: FIGO stage at diagnosis (p=0.0168), ovarian cancer type (p=0.0166), residual tumor size (p=0.0026), tau expression status (p=0.0198) (Figure 3) and sensitivity to first-line chemotherapy (p<0.0001). Age, performance status and tumor grade were not correlated with OS.

**Table 4:** Univariate analysis of OS correlation with clinical parameters (log-rank test)

**Clinical parameter**	**n (% )**	**Median (months)**	**P value**
Age			0.5287
o < 65	60 (81.1%)	41.8	
o > 65	14 (18.9%)	36.6	
FIGO stage at diagnosis			
o Early (I,II)	15 (20.3%)	88.2%†	0.0168*
o Advanced (III,IV)	59 (79.7%)	50.5%†	
Histopathologic cell type			
o serous	37 (50%)	33.4	0.0166*
o others	37 (50%)	54.8	
Residual tumor size			
o <1 cm	48 (64.9%)	50.2	0.0026*
o > 1 cm	26 (35.1%)	22.6	
Performance status (ECOG)			
o 0-1	69 (93.2%)	42.9	0.3461
o 5 (6.7%)	5 (6.7%)	15.1	
Tumor grade			
o G1,G2	31 (41.9%)	49.0	0.2099
o G3, unknown	43 (58.1%)	30.0	
Sensitivity to first-line chemotherapy			
o Resistant (<6 months)	26 (35.1%)	4.6%†	<0.0001*
o Sensitive (>6 months)	48 (64.9%)	87.8%†	
Tau expression			
o negative	19(25.6%)	80.2%†	0.0198*
o positive	55(79.3%)	52.4%†	

†− if median was not achieved, the results were described as a percentage of patients with 3-year OS.

*- statistical significance.

In multivariate analysis only sensitivity to first-line chemotherapy remained statistically significant (p<0.0001, HR-22.59) as an independent parameter associated with OS (Table 5).

**Table 5:** Multivariate analysis of OS association with clinical parameters

**Clinical parameter**	**Multivariate analysis**	
	**HR (95% CI)**	**P value**
Tau expression	NS	NS
▪ negative		
▪ positive		
FIGO stage at diagnosis	NS	NS
○ Early (I,II)		
○ Advanced (III,IV)		
Histopathologic cell type	NS	NS
○ serous		
○ others		
Residual tumor size	NS	NS
○ <1 cm		
○ > 1 cm		
Sensitivity to first-line chemotherapy	**22.59; 95% CI, 8.71-58.55**	**<0.0001**
○ Resistant (<6 months)		
○ Sensitive (>6 months)		

### Association between Tau expression and response to chemotherapy in patients with measurable disease

Among 46 patients with measurable target lesions, 11 (23.9%) were assessed as Tau-negative and 35 (76.1%) as Tau-positive. Proportion of objective response (OR) was higher in Tau-negative group (90.9%) compared with Tau-positive group (54.3%), with statistical significance (p=0.0299). The results are demonstrated in Table 6.

**Table 6:** Association between Tau expression and response to chemotherapy in patients with measurable target lesions according to RECIST scale (n=46)

**Response to chemotherapy according to RECIST**	**Negative Tau expression (n=11)**		**Positive Tau expression (n=35)**		**Mann – Whitney test U**	
	**n**	**%**	**n**	**%**	**Z**	**P**
OR (CR+PR)	10	90.9%	19	54.3%	2.17	0.0299
SD+PD	1	9.1%	16	45.7%		
CR	10	90.9%	18	51.4%	2.09	0.0362
PR	-	-	1	2.9%		
SD	-	-	9	25.7%		
PD	1	9.1%	7	20%		

Abbreviations: OR – objective response, CR – complete response, PR- partial response, SD – stable disease, PD – progression disease.

## Discussion

Currently, the most effective chemotherapy in ovarian cancer, recognized as a gold standard is platinum analogue combined with paclitaxel. About 70% of the patients respond to this regimen. The others potentially could benefit from different drugs. However, no predictive factors are known in ovarian cancer.

As far as we are concerned, in our study Tau protein was assessed in the tissues of ovarian cancer for the first time by the use of immunohistochemistry (IHC). Majority of the patients was acknowledged as Tau-positive (74.3%), while 25.6% of the patients was Tau-negative. The results differ from those achieved in other studies. Rouzier et al. recognized 52% of the breast cancer patients as Tau-negative [4]. Similar proportion (57% of Tau-negative) was demonstrated by Pusztai et al. [8] 30% of the patients with gastric cancer in Mimori et al. study was identified as Tau-negative [9]. Obtained findings indicate that Tau protein expression might differ among cancer sites.

In our study, Tau-negative status in primary tumor of ovarian cancer was identified as a predictive factor for paclitaxel-containing chemotherapy. Both groups seem to be well balanced regarding to age, FIGO stage, histological type, performance status and grade (Table 7) so it does not seem that there were any biases in this field although it necessary to remember that our study was conducted retrospectivly, so its value is limited. In univariate analysis median PFS was 12.8 months longer in Tau-negative group (p=0.0355). Among 46 patients with measurable target lesions, those qualified as Tau-negative achieved statistically significant more objective responses according to RECIST criteria in comparison to patients with Tau-positive ovarian cancers (90.9% and 54.3% respectively; p=0.0299).

**Table 7:** Clinicopathologic features in ovarian cancer patients according to Tau expression

**Clinical parameter**	**Negative Tau expression**	**Positive Tau expression**	**p value**
Age			
o < 65	16	44	p= ,8496*
o ≥ 65	4	10	
FIGO stage at diagnosis			
o Early (I,II)	7	10	p= ,1345
o Advanced (III,IV)	13	44	
Histopathologic cell type			
o Serous	8	29	p= ,2951
o others	12	25	
Performance status (ECOG)			
o 0-1	19	50	p= ,8768*
o 2	1	4	
Tumor grade			
o G1,G2	12	19	p= ,0547
o G3, unknown	8	35	

Abbreviations: p- Probability values calculated by the Chi-square test,

* p- Values calculated by the Chi-square test with Yates correction.

Thus, negative status of Tau in primary tumor of ovarian cancer is associated with better efficacy of chemotherapy. It may result from paclitaxel’s action, competitive to Tau protein. Paclitaxel binds beta-tubulin on microtubule’s inner surface, in the same point as Tau protein [5]. It leads to inhibition of depolimerisation process, interferes with spindle function and hinders cell division [6]. Presence of Tau protein on the microtubules’ surface creates difficulties in paclitaxel combining to these structures. Low Tau expression may result in better paclitaxel connection with microtubules and more effective chemotherapy action, expressed in higher objective responses rate and better PFS.

So far, predictive role of Tau expression was assessed in breast and gastric cancers. Low Tau protein expression (Rouzier) or low Tau-mRNA (Andre) was associated with statistically significant more frequent achievement of complete response (CR) to paclitaxel in breast cancer. Similarly, in the study of Tanaki et al. [10], significantly more Tau-negative patients with metastatic breast cancer benefited from paclitaxel therapy, compered with Tau-positive group of patients.

However, about half of Tau-negative patients receiving paclitaxel was not sensitive to this chemotherapy[4,10]. The other mechanisms of resistance to paclitaxel: tubulin mutations, different tubulin isoforms, overexpression of multidrug resistance proteins or bcl-2 might be responsible for this phenomenon. Identification of single factor (in our case Tau protein expression) might not be sufficient to provide selection to the certain treatment.

While *in vitro* down-regulation of Tau gene by anti-Tau siRNA in paclitaxel-resistant cell lines caused increase of their sensitivity to this drug, inhibition of Tau protein may enhance paclitaxel activity [4].

Predictive value of Tau protein was not confirmed in some studies [8,11-13]. Statistically non-significant trend of increased sensitivity to paclitaxel was observed in Tau-negative ER(−) breast cancer patients [11]. ER(−) and ER(+) patients should be analyzed separately. Function of Tau gene is regulated by estrogens and expression of Tau protein *in vitro* might be induced by these hormones as well as tamoxifen. Additionally, predictive value of low Tau expression for paclitaxel therapy was confirmed in gastric cancer, potentially hormone-independent malignancy [9].

The results of the recent study of Fekete et al. reveal a possible prediction of relapse-free survival by Tau gene expression by TaqMan Real Time Polymerase Chain Reaction (RT-PCR) and relapse-free survival [14].

Prognostic value of Tau expression in ovarian cancer patients treated with paclitaxel and platinum-based chemotherapy was revealed in univariate analysis of our study. Percentage of patients achieving 3-year overall survival was significantly higher in Tau-negative group (80.2%) than in Tau-positive (52.4%).

Our results differ from those obtained in the studies on breast cancer, where co-expression of Tau protein and estrogen receptor was considered as good prognostic factor [8,11,15]. This divergence might be caused by Tau significance evaluation in different cancer sites. Hormone-dependent breast cancer is associated with good prognosis and chemo resistance. Tau genes are regulated by estrogens and tamoxifen so Tau protein expression is associated with hormones. On the other hand, in ER-negative breast cancer patients group prognostic value of Tau protein was not confirmed. In other study prognostic value of Tau protein in breast cancer was not observed [13].

The only independent parameter significantly influencing on OS in multivariate analysis was sensitivity to first-line chemotherapy (HR 22.59; p<0.0001), defined as no progression or recurrent disease in 6 months from the end of treatment. The aim of adjuvant chemotherapy is prolongation of OS as well as PFS. The effect is possible to achieve if malignancy is prone to drugs. Thus, chemosensitivity is a good prognostic factor.

## Conclusions

Many studies confirm prognostic value of time duration between chemotherapy ending and disease progression in ovarian cancer [16-18]. Extension of this period might be caused by tumor susceptibility to cytostatics as well as maximal cytoreduction during surgery.

Mechanisms affecting chemosensitivity are complex. Tau expression is a single factor, influencing sensitivity to paclitaxel. Platinum analogue (the other component of standard regimen in ovarian cancer) effectiveness is modified by numerous factors such as epigenic changes in cancer cells, expression of multidrug resistance proteins (for example: P-gp, MRP1, MRP2, LRP), p53 gene mutations and GST-pi increase [19]. The processes are intricate, thus identification of single factors seems to be complicated, especially in polichemotherapy.

Better response to paclitaxel related to negative status of Tau protein in primary tumors in ovarian cancer is conducive to extension of PFS, and therefore to the improvement of prognosis in ovarian cancer patients. Although sample size in our analysis was not great and the data were evaluated retrospectively, the results of our study may direct successive researches in ovarian cancer. Significance of Tau protein expression requires evaluation in prospective studies with larger group of patients, including assessment of the other predictive and prognostic parameters in paclitaxel and platinum-based chemotherapy.

## Competing interests

The authors declare that they have no competing interests.

## Authors’ contributions

MS participated in the design of the study, collected data, prepared specimens for staining, analyzed the results and drafted the manuscript. LB participated in the design of the study and performed the statistical analysis. BG and KZ carried out the immunochemistry staining and assessed the slides. RS helped to analyze the data and draft the manuscript. WK helped to analyze the data. CS participated in the study design and coordination, revised the manuscript critically and gave final approval of the version to be published. All authors read and approved the final manuscript.
